# The *Escherichia coli *K-12 ORFeome: a resource for comparative molecular microbiology

**DOI:** 10.1186/1471-2164-11-470

**Published:** 2010-08-11

**Authors:** Seesandra V Rajagopala, Natsuko Yamamoto, Adrienne E Zweifel, Tomoko Nakamichi, Hsi-Kuang Huang, Jorge David Mendez-Rios, Jonathan Franca-Koh, Meher Preethi Boorgula, Kazutoshi Fujita, Ken-ichirou Suzuki, James C Hu, Barry L Wanner, Hirotada Mori, Peter Uetz

**Affiliations:** 1 J Craig Venter Institute (JCVI), 9704 Medical Center Drive, Rockville, MD 20850, USA; 2 Graduate School of Biological Sciences, Nara Institute of Science and Technology, Ikoma, Nara, Japan; 3 Dept. of Biochemistry and Biophysics, Texas A & M University, College Station, TX 77843-2128, USA; 4 National Institute of Technology and Evaluation, Kazusakamatari, Kisarazu, Chiba 292-0818, Japan; 5 Department of Biological Sciences, Purdue University, West Lafayette, IN 47907, USA; 6 Institute for Advanced Biosciences, Keio University, Tsuruoka City, Yamagata, Japan; 7 Proteros Biostructures, Martinsried, Germany

## Abstract

**Background:**

Systems biology and functional genomics require genome-wide datasets and resources. Complete sets of cloned open reading frames (ORFs) have been made for about a dozen bacterial species and allow researchers to express and study complete proteomes in a high-throughput fashion.

**Results:**

We have constructed an open reading frame (ORFeome) collection of 3974 or 94% of the known *Escherichia coli *K-12 ORFs in Gateway^® ^entry vector pENTR/Zeo. The collection has been used for protein expression and protein interaction studies. For example, we have compared interactions among YgjD, YjeE and YeaZ proteins in *E. coli*, *Streptococcus pneumoniae*, and *Staphylococcus aureus*. We also compare this ORFeome with other Gateway-compatible bacterial ORFeomes and show its utility for comparative functional genomics.

**Conclusions:**

The *E. coli *ORFeome provides a useful resource for functional genomics and other areas of protein research in a highly flexible format. Our comparison with other ORFeomes makes comparative analyses straighforward and facilitates direct comparisons of many proteins across many genomes.

## Background

High-throughput DNA sequencing has increased the number of genome sequences to over 1,000 bacterial species from which we can infer their proteomes and often major parts of their metabolism and regulatory pathways. A systems level understanding of cells, however, will require the functional characterization of these proteins and how they work together. In recent years, a growing number of efforts have used high throughput assays to catalog gene expression, protein interactions, localization and metabolic activities. For many of these studies, the first step is to identify and then clone all the open reading frames (the "ORFeome") encoded by the genome of the organism [1].

Here we describe the construction of a comprehensive *Escherichia coli *ORF collection in a Gateway^® ^[2] entry vector. The library represents 3974 ORFs or 94% of all protein-coding genes. The Gateway^® ^system facilitates the transfer of ORFs into a large range of expression vectors that are suitable for downstream studies. Here we demonstrate the utility of the *E. coli *ORFeome by comparing it to 12 other available microbial ORFeomes and by testing a set of protein-protein interactions among 5 species.

**Figure 1:**
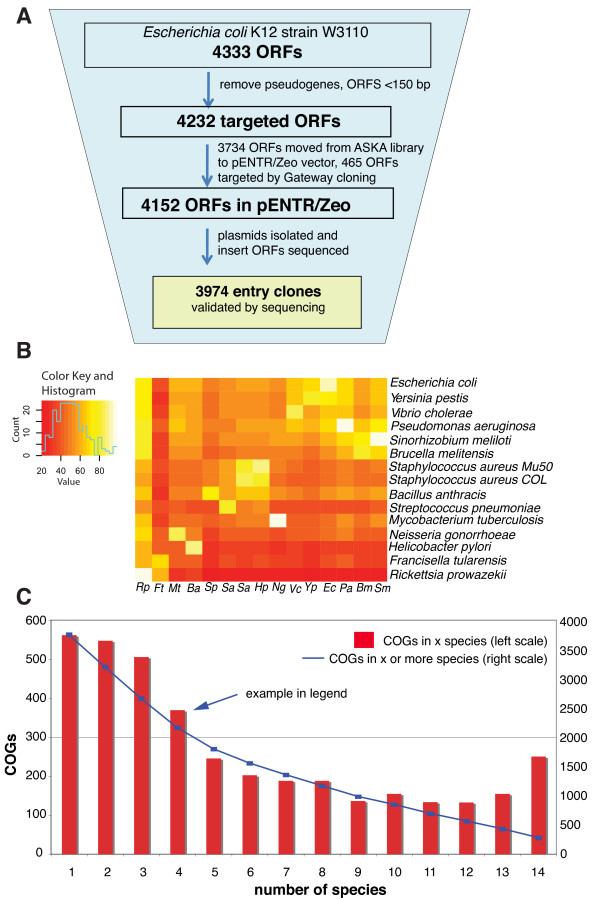
***E. coli *entry clone libray construction and comparative ORFeomics**. **(A) **Pipeline used to clone the *E. coli *ORFs into Gateway^® ^Entry vector (pENTR/Zeo). The total number of ORFs in common between *E. coli *K-12 MG1655 and *E. coli *K-12 W3110 is based on the more accurate sequencing of these strains [14] and community re-annotation [3]. (**B**) Pairwise comparison of Clusters of orthologous genes (COGs) in all species pairs. Colors indicate the similarity between species. For example, *E. coli *shares at least 60% of its COGs with most other species. The species in the rows are ordered such that similar rows are near each other. **(C) **Presence of COGs in 14 species with available ORFeomes. For example, 370 COGs are present in exactly 5 species (bars and left scale). The line represents the number of COGs that are present in a minimal number of species, e.g. 2162 COGs are present in 4 or more species (right scale).

The complete genome sequence of *Escherichia coli *K-12 encodes 4333 protein-coding ORFs [3] (http://cmr.jcvi.org/). Kitagawa et al. previously cloned all the *E. coli *ORFs (the "ASKA library") into an expression vector creating N-terminal 6xHis and C-terminal GFP fusions [4]. However, the ASKA library cannot be used to flexibly transfer ORFs into other expression vectors [5,6]. Libraries of all open reading frames cloned into highly flexible vectors will be needed to take full advantage of the information found in any genome sequence. We transferred the ASKA library [4] into an Gateway^® ^entry vector (pENTR/Zeo) by *Sfi*I restriction enzyme cloning (Figure 1). About 250 *E. coli *clones which were not present in the ASKA library or which were not successfully cloned from the ASKA library into the Gateway^® ^entry vector were cloned directly by Gateway^® ^recombination (see **Methods**). The entry clone library was then validated by DNA sequencing. The resulting library represents 3974 ORFs (Additional file 1, **Table S1**). The clone collection is freely available to academic users.

The *E. coli *entry clone library lacks start and stop codons and is thus compatible with both N-terminal and C-terminal expression clone constructions. The clones from the entry vectors can be easily shuttled into different Gateway-compatible expression vectors of many types in a high-throughput fashion [5,6].

## Results and Discussion

### *E. coli *as a model for comparative genomics and biology

*E. coli *K-12 has led basic life science research for more than half a century due to its easy manipulation and its safety as a non-pathogenic organism. We wondered to what extent it can also serve as model for pathogenic bacteria and compared the *E. coli *ORFeome to all other bacterial ORFeomes that are available as Gateway-compatible clones. Figure 1b shows how many *E. coli *genes have orthologs in these species including *Vibrio cholerae, Yersinia pestis, Streptococcus pneumoniae *and others. For example, over 80% of *E. coli *COGs are conserved in *Pseudomonas aeruginosa *(Figure 1b). COGs (clusters of orthologous groups) represent conserved protein families and provide a standard way to compare gene sets [7]. We can safely assume that the general molecular function of these *E. coli *proteins should be similar or identical to these homologues in other bacterial species. However, we cannot easily predict whether small changes in sequence will change the function or specificity of proteins. The availability of complete collections of easily moveable cloned ORFs facilitates functional studies in multiple species in parallel, even at the level of whole proteomes. As of today, Gateway^® ^clone collections are available for at least 14 bacterial species including 2 strains of *Staphylococcus aureus *(Figure 1B,C, Additional file 1, **Table S3**). COGs should also facilitate comparative analysis, given that many of them are present in species for which ORFeomes are available. For example, 2162 COGs are present in at least four of the species for which ORFeomes are available (Figure 1C).

### The *E. coli *ORFeome for protein expression

**Figure 2:**
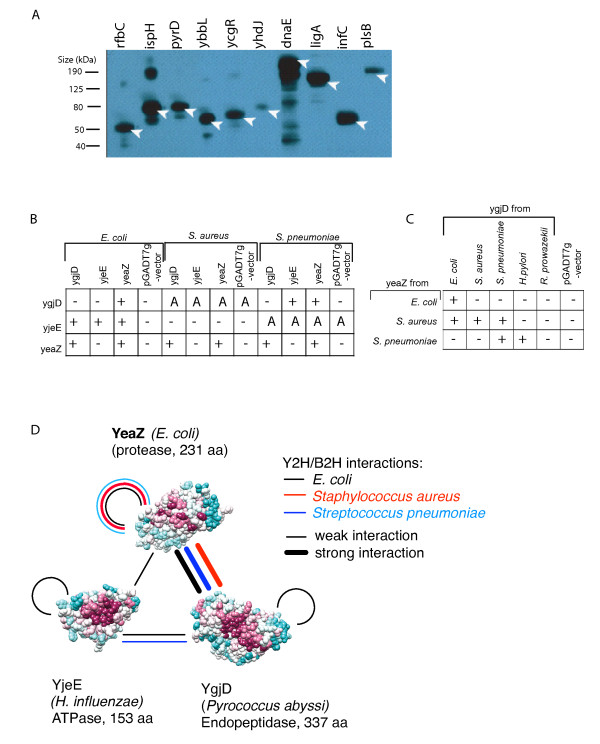
**Functional ORFeomics**. **(A) **Recombinant protein expression in *E. coli*. Western blot of 10 GST-tagged *E. coli *recombinant proteins. After induction of protein expression with IPTG, the cells were lysed and the crude lysates were analyzed by Western blot and antibody detection with anti-GST antibodies. The expected band sizes are marked by arrow heads, the lower molecular weight bands of DnaE are the results of protein degradation **(B, C) ***E. coli*, *S. aureus *and *S. pneumoniae *YgjD, YjeE and YeaZ proteins were tested by the yeast two-hybrid method for interactions. "+" indicate a positive protein interaction and "-" indicate no protein interaction; the pGADT7g-vector is a negative control (for autoactivation); uncertain interactions due to autoactivation are indicated by "A". **(C) **Intraspecies and interspecies protein interactions of YgjD and YeaZ of *E. coli*, *S. aureus*, *S. pneumoniae, H. pylori*, and *R. prowazekii *(as in **B**). **(D) **Interpretation of Figures (B) and (C). Crystal structures are available for YeaZ (PDB: 1OKJ), YjeE (1HTW-A), and YgjD (2IVO), but only YeaZ has been crystallized from *E. coli*. Comparative analyses show conserved residues and thus potential interaction sites (red: most, blue: least conserved). The strongest interactions (thick lines) also tend to be the most conserved ones.

We verified the functionality of the entry clones by two different downstream applications. First, we tested recombinant protein production in *E. coli *by randomly selecting ten entry clones from the library that were subsequently cloned into the Gateway^® ^GST-fusion expression vector pDEST-GST (see **Methods**). The expression clones were transformed into the BL21(DE3) protein expression strain of *E. coli *and after induction of protein expression with IPTG, the cells were lysed in sample buffer and analyzed by Western blot (Figure 2A). Second, we tested protein interactions by the yeast two hybrid assay. About 90 entry clones which are known to be involved in bacterial motility (flagellum) and chemotaxis were cloned into two different yeast two-hybrid expression vector systems and 90×90 = 8,100 protein pair were tested for protein-protein interactions, resulting in 177 protein-protein interactions [6].

### The *E. coli *ORFeome for functional genomics and protein interaction analysis

The availability of ORFeome collections will greatly facilitate comparative functional genomic studies. An example of this is to compare protein-protein interactions among multiple species in order to determine which interactions are conserved. Here we used the *E. coli *ORF collection as well as previously generated *S. aureus *and *S. pneumoniae *collections (http://pfgrc.jcvi.org/) to systematically test, by yeast two-hybrid, whether the recently described protein-protein interactions between the essential *E. coli *gene products YgjD, YjeE and YeaZ are conserved in these Gram-positive pathogens. These three proteins were selected as an interesting case study because they are highly conserved, essential, and of unknown function. The *yjeE*, *yeaZ*, and *ygjD *genes are highly conserved throughout eubacterial genomes while *ygjD *orthologs are also found throughout the archaea and eukaryotes. We found all the interactions that Handford et al. [8] reported but there were significant differences between species (**Figure B, C, D**). For example, YjeE and YeaZ from *E. coli*, but not from *S. aureus *or *S. pneumoniae*, interacted. The functions of these genes remain poorly understood. In *E. coli*, yeaZ is able to proteolyse ygjD while yjeE, an ATPase, competes with ygjD for binding to yeaZ. The inability of yjeE to interact with yeaZ *in S. aureus *and *S. pneumoniae *may indicate differences in the regulation of the ygjD-yeaZ complex in these species. Our study of these interactions not only demonstrated differences between the species tested but also showed another advantage of such a comparative approach: the *S. pneumoniae *YjeE as well as the the *S. aureus *YgjD protein autoactivated the reporter genes when fused to the Gal4 DNA binding domain. This property affects approximately 10% of bait proteins in yeast two-hybrid assays [9]. However, while the *S. aureus *YgjD bait is autoactivating, YgjD of *E. coli *and *S. pneumoniae *are not (Figure 2B). Hence, comparative assays may offer one strategy for circumventing limitations of the yeast two-hybrid method.

Additionally, by revealing which interactions are evolutionarily conserved, such comparative studies will greatly enhance our ability to interpret the conserved biological functions of the interacting proteins, and also the computational analysis of high-throughput protein-protein interaction datasets [10]. For example, crystal structures are available for all three interacting proteins, but only one from *E. coli*, namely YeaZ (Figure 2D). In order to obtain more information for model building and functional interactions, we expanded our test set beyond *E. coli *and tested the interactions among YgjD, YeaZ, and YjeE in five different species, including *H. pylori *and *R. prowazekii*. In addition to the expected *intra*species interactions, *inter*species interactions were observed (Figure 2C). The *S. aureus *YeaZ protein associated with the products of the *E. coli *and *S. pneumoniae *orthologs of *ygjD *while the *S. pneumoniae *YeaZ protein was able to interact with *H. pylori *YgjD. This last interaction was particularly unexpected as *yeaZ *is not conserved in *H. pylori*, and suggests the possibility that the functions of *yeaZ *may be performed by another protein in this species.

Given the availability of ORFeomes for more than a dozen species, such comparative analyses can now be carried out quite easily. More importantly, additional biochemical or genetic studies can be done in *E. coli *for which extensive resources, including deletion strains [11,12] and comprehensive databases (http://www.PrFEcT.org > Resources), are available. For instance, our *E. coli *clones could be used to complement mutants in other species, which would demonstrate their functional equivalence.

## Conclusions

In conjunction with other clone sets and the vast amount of genomics and proteomics data from *E. coli*, the Gateway-ORFeome will be another highly useful resource for the *E. coli *functional genomics community.

## Methods

### Cloning the *E. coli *ORFs from ASKA library to Gateway^® ^entry vector

**Figure 3:**
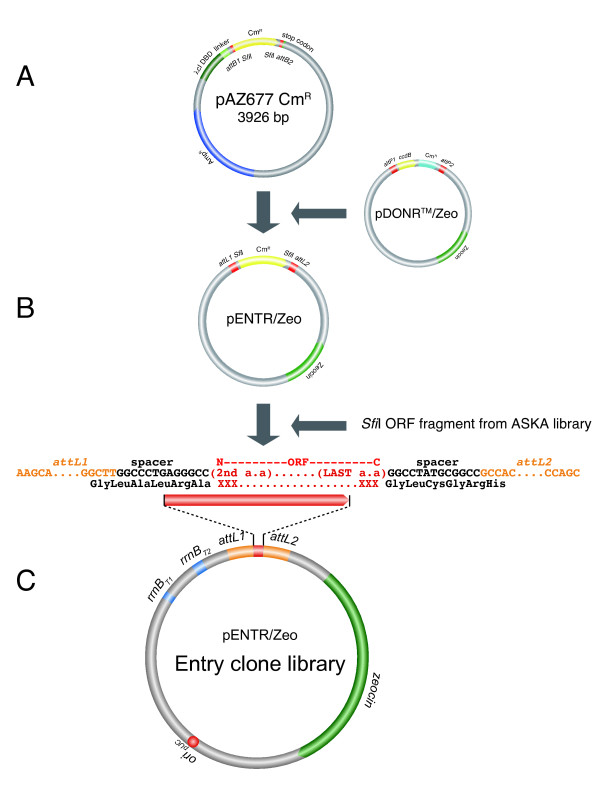
**Vectors and cloning strategy**. (**A**) pAZ677 Cm^R^. (**B**) Construction of pENTR/Zeo by BP recombination with pDONR™/Zeo. The resulting vector has attL1, attL2 sites and two SfiI sites bordering the Cm^R ^fragment. (**C**) The *E. coli *ORFs of pCA24N were then transferred by SfiI digestion, gel-fractionated and ligated into SfiI-digested pENTR/Zeo vector. The positive clones are selected for Zeocin resistance and Chloramphenicol sensitivity, and are validated by PCR and DNA sequencing (see methods).

In order to transfer the ASKA library [4] into the Gateway^® ^entry vector pENTR/Zeo, the ASKA library plasmids (in pCA24N) were first digested with SfiI to release the ORFs. The purified ORFs were then ligated into the SfiI-digested pENTR/Zeo vector (Figure 3C). The ligation reaction was then transformed into *E. coli *DH5-alpha competent cell and plated on LB-Zeocin agar plates. We picked 4 isolated colonies for each reaction and verified them for the chloromphenicol antibiotic sensitivity and the size of insert by colony PCR. The correct clones were re-arrayed and the plasmids isolated. The insert ORFs were verified by DNA sequencing (see sequence validation).

About 250 *E. coli *clones which were not present in the ASKA library or not successfully ligated from the ASKA library into the Gateway^® ^entry vector were cloned by Gateway^® ^recombinational cloning [2]. The PCR products were inserted into the Gateway^® ^entry vector pDONR™/Zeo (Invitrogen) by BP-cloning. The products resulting from site-specific recombination were transformed into *E. coli *and plated onto solid LB medium containing Zeocin. Two isolated colonies were selected for each reaction and the clones were verified by colony-PCR with pDONR™/Zeo-specific primers. The clones that had an insert of the expected size were picked for plasmid isolation and the plasmid was used as a template for DNA sequencing to verify the insert sequence.

### Colony PCR of bacterial clones

We selected four isolated colonies for each pENTR/Zeo clone to verify the cloned ORF size. Colonies were picked with a sterile pipette tip and transferred to the wells of a 96-well plate containing 150 μl low-salt LB liquid medium containing 50 μg/ml Zeocin™ and incubated overnight at 37°C to generate glycerol long-term frozen stocks. 1 μl of bacterial culture was used for colony PCR in 96-well plates containing 50-μl samples with Biomix™ (Bioline, Cat. No. BIO-25012), pDONR™/Zeo-specific forward primer (5'-GTAAAACGACGGCCAG-3') and reverse primer (5'-CAGGAAACAGCTATGAC-3') (0.3 μM each). The 30 PCR cycles (94°C for 30 s, 55°C for 30 s, and 72°C for 1 min/kb) were preceded by heating to 94°C for 5 min and followed by a 7-min incubation at 72°C. The sizes of the PCR products were determined by agarose gel electrophoresis and ethidium bromide staining.

### Primer design

The DNA sequence of *E. coli *strain K-12 was obtained from the JCVI/CMR Genome Database (http://cmr.jcvi.org/cgi-bin/CMR/CmrHomePage.cgi) and primers were designed for clones which were cloned by Gateway^® ^recombination (Figure 3), using the primer design tool (http://tools.bio.anl.gov/bioJAVA/jsp/ExpressPrimerTool/). The primers are designed without endogenous start and stop codons. In addition to a 20- to 30-nucleotide-long ORF-specific sequence the *attB1 *segment (5'-aaaaagcaggctta-3') was added to each forward primer, followed by ORF-specific bases without a start codon. The *attB2 *segment (5'-agaaagctgggtg-3') was added at the 5' end of each reverse primer, which was complementary to the end of the ORF, without the last nucleotide of the stop codon. The primers were obtained from Invitrogen in a 96-well format.

### PCR amplification of the ORFs

For the clones constructed by Gateway^® ^recombinational cloning, PCR was performed in 96-well plates containing 50-μl reaction volumes consisting of 1 U KOD DNA polymerase (Novagen), dNTP mix (0.4 mM each), primary forward and reverse primers (0.3 μM each), and ***E. coli K-12 *strain W3110 **genomic DNA (200 ng). The complete sequences of *attB1 *(5'-GGGGACAAGTTTGTACAAAAAAGCAGGCT-3') and *attB2 *(5'-GGGGACCACTTTGTACAAGAAAGCTGGGT-3') were added in the secondary round PCR, where the first round PCR product was used as a template, to generate the full-length *attB1 *and *attB2 *sites flanking the ORFs. The PCR cycles were used as recommended by the KOD DNA polymerase manufacturer (Novagen, Cat. No.710853). These PCR products were used for BP reaction.

### *attB × attP *recombination reactions (BP reactions)

The PCR-amplified ORFs with *attB1 *and *attB2 *sites were recombined into the vector pDONR™/Zeo (Invitrogen) by using the BP Clonase™ II Enzyme Mix (Invitrogen). In 96-well plates, samples containing 1 μl purified PCR product, 1 μl BP Clonase™ II Enzyme Mix, 75 ng pDONR™/Zeo plasmid and TE buffer, pH 8.0, up to 5 μl were incubated overnight at 25°C. After adding 1 μg proteinase K (Invitrogen) and incubating at 37°C for 30 minutes, the BP reactions were directly used for bacterial transformation.

### *attL × attR *recombination reactions (LR reactions)

Entry vectors were set up in LR reactions to recombine the gene of interest into several destination vectors (expression vectors). The destination vectors used were pDEST22, pDEST32 (Invitrogen), pGADT7g, pGBGT7g and pDEST-GST vectors. Samples containing 5 μl prepared entry clone, 1 μl LR Clonase™ II Enzyme Mix (Invitrogen), destination vector (150 ng), and TE buffer, pH 8.0 to 10 μl were incubated at 25°C for two hours. After adding 1 μg proteinase K (Invitrogen) and incubating at 37°C for 30 min, the LR reactions were directly used for plasmid transformation into *E. coli*.

### Validation of entry clones by DNA sequencing

To sequence verify the inserted ORFs we re-arrayed each clone which showed the right size as a PCR product. All these clones were grown on 1.2 ml LB-Zeocin liquid medium in 96 deep well plates (2 ml Qiagen). An aliquot of this culture (50 ul) was used to make a glycerol stock for longer storage. The plasmid DNA was isolated by using 96 well plasmid preparation plates (Millipore), and the plasmid preparations were sequenced with a pDONR™/Zeo-specific forward primer and reverse primers to verify the insert from both N-terminal and C-terminal ends of the ORFs. All the sequencing reads were analyzed using NCBI stand alone BLAST against the *E. coli K-12 *genome database to confirm the identity of each ORF. The clone verification was classified into three categories based on the sequencing coverage of the insert, **class A**: insert is verified from both N and C-terminal ends; **class B**: insert is verified either from N or C-terminal ends (Additional file 1, **Table S1**); **class C**: unverified (or sequence failed).

### Validation of entry clones by recombinant protein production

In order to verify the functionality of the ORFeome, a random sample was used to show expression of proteins from the cloned ORFs. Ten different *E. coli *entry clones were shuttled into the pDEST-Exp vector designed to make a fusion protein with a GST-Tag. The resulting expression vectors were transformed into the *BL21*(DE3) protein expression strain of *E. coli*. After induction of protein expression with IPTG, the cells were lysed and the crude lysates were analyzed by western blot and antibody detection anti-GST antibodies. Protein was expressed from all 10 of the GST-tagged proteins we tested (Figure 2A).

### Yeast two-hybrid analysis

The yeast two-hybrid assay is conducted as described by Rajagopala et al. (Rajagopala et al. 2007b).

### Homology of bacterial ORFeomes

The cluster of orthologous group (COGs) for all the 14 bacterial species for which cloned ORFeomes are available (Additional file 1, **Table S3**), were extracted from the STRING database [13]. This table was used to obtain orthologous protein information between different bacterial species based on COGs relationship. The COGs from *Escherichia coli *K-12 (strain W3110) were used as reference to obtain homologous proteins in 14 different bacterial species (including two strains of Staphylococcus aureus, Col and Mu50; only Col was used for the COG analysis though). Similarly, by taking each of these 14 species as reference, the homologues for the rest of the species were extracted. Unique proteins from each species in the COGs were taken and the fraction of these out of all the predicted protein coding genes of the respective genome was used to calculate the percentage of homologous proteins. The matrix with all these values with reference as well as other species was made and used to generate a heat map in order to represent the percentage of homologous proteins in different species (Figure 1B).

## Authors' contributions

SVR, NY, TN, JDMD, and HKH carried out all cloning reactions and sequence-verified the clones. AEZ made the pENTR/Zeo vector. JFK analyzed the interactions among YeaZ, YgjD, and YjeE. MPB carried out the COG analyses. JCH, BLW, HM, and PU conceived, designed, and supervised this project. SVR, BLW, and PU wrote the manuscript. All authors read and approved the final manuscript.

## Supplementary Material

Additional file 1 **Supplementary Tables S1-S5**. S1: All *E. coli *ORFs; S2: ORFs not targeted; S3: list of available ORFeomes; S4: Yeast two-hybrid interactions; S5: homologous ORFs in other species organized by COGs (clusters of orthologous groups/genes). Click here for file
